# TRANSMISSION OF RELIGIOSITY BETWEEN GRANDPARENTS AND GRANDCHILDREN AS A BASIS FOR ELDERCARE RESPONSIBILITY

**DOI:** 10.1093/geroni/igad104.1744

**Published:** 2023-12-21

**Authors:** Merril Silverstein, Seonhwa Lee, RianSimone Harris, Wencheng Zhang

**Affiliations:** Syracuse University, Syracuse, New York, United States; Syracuse University, Syracuse, New York, United States; Syracuse University, Syracuse, New York, United States; Syracuse University, Syracuse, New York, United States

## Abstract

Different branches of research demonstrate that religiosity is effectively transmitted from grandparents to grandchildren and that religiosity strengthens norms of eldercare responsibility. This paper unites these two branches of inquiry by proposing that eldercare norms in emerging adulthood are directly and indirectly formed through an intergenerational religious pathway. Data derive from 149 grandparent-grandchild dyads from the 2021-22 wave of the Longitudinal Study of Generations. Religiosity is defined as a composite consisting of self-assessments of religious importance, religious intensity, and religious service attendance. Eldercare norms are assessed based on an evaluation of the obligation that adult children should have to provide support and care to older parents in six areas of assistance. Intermediate factors include grandchildren’s familistic values and emotional closeness to grandparents. Structural equation modelling was used to examine direct and indirect pathways linking these factors. Results indicate significant continuity in religiosity between grandparents and grandchildren. The impact of grandchildren’s religiosity on their eldercare norms is fully mediated by their familistic values and emotional closeness with grandparents. This analysis reveals that the transmission of religiosity to grandchildren begets eldercare norms through an ideational route of strengthening values of familism, and a personal route of promoting closer, and possibly more empathetic, relationships with grandparents who have or may soon have support needs. Results demonstrate the “hidden” impact of religious orientations of older grandparents on grandchildren and serve as a counterpoint to potentially specious reports about the adverse impact of declining religiosity and weakening intergenerational cohesion on aging families in American society.

